# The American Microscopical Society

**Published:** 1893-04

**Authors:** 


### The American Microscopical Society.

We have received a special circular referring to the fifteenth annual meeting of the American Microscopical Society, in which the writer has been interested since its organization. The list of papers presented at the meeting includes a large number of highly interesting scientific dissertations on questions of an eminently practical character, as well as those of technical value. A large number of new and valuable microscopical appliances and accessories were exhibited. A question was raised whether it would not be wise to hold the meetings of the Society at the same time and place as the American Society for the Advancement of Science. The fact that many of the members of each Society are also members of both Societies would seem to indicate the wisdom of this suggestion. The American Microscopical Society has done much to place the science of microscopy in this country upon a footing with the work done by microscopists of England and continental Europe, and we are pleased to note that the Society seems to be in a flourishing condition.

